# Longitudinal Effects of a Nutritional Intervention and Exercise Program on Trunk Muscle Mass in Older Adults: A Six-Month Preliminary Longitudinal Study

**DOI:** 10.7759/cureus.88330

**Published:** 2025-07-19

**Authors:** Yutaro Hyodo, Takumi Jiroumaru, Kenji Mori, Tomoka Hattori, Ikkei Tanaka, Takamitsu Fujikawa

**Affiliations:** 1 Department of Physical Therapy, Bukkyo University, Kyoto, JPN; 2 Rehabilitation, Kanazawa Orthopaedic and Sports Medicine Clinic, Ritto, JPN

**Keywords:** nutrition rehabilitation, older adults, skeletal muscle mass, trunk muscle mass, sarcopenia

## Abstract

Objective: This preliminary study aimed to investigate six-month longitudinal changes in upper limb, lower limb, and trunk muscle mass, as well as gait speed and handgrip strength, in older adults receiving a nutritional intervention and rehabilitation.

Methods: This longitudinal study included 18 community-dwelling older adults (age 80.4 ± 3.8 years) attending a daycare facility. Participants received a nutritional supplement drink and an individualized exercise program consisting of resistance training and cycle ergometer exercise. Upper limb, lower limb, and trunk muscle mass (using bioelectrical impedance analysis), maximal gait speed, and handgrip strength were measured at baseline, three months, and six months. Data were analyzed using a repeated measures analysis of variance (ANOVA).

Results: A repeated measures ANOVA showed significant change over time in both trunk muscle mass and upper limb muscle mass (p < 0.05). Post hoc tests confirmed that the increase in trunk muscle mass was significant between baseline and six months, whereas no significant pairwise differences were found for upper limb muscle mass. No significant changes were observed for the other variables.

Conclusion: This preliminary study suggests that a six-month combined nutritional and exercise intervention may significantly increase trunk muscle mass in older adults requiring daycare. These findings highlight the potential importance of targeting trunk muscles in rehabilitation nutrition strategies and warrant further investigation in larger, controlled studies.

## Introduction

Nutritional intake in older adults has garnered increasing attention with the rising prevalence of sarcopenia. Rehabilitation nutrition is an integrated approach combining nutritional management with rehabilitation to restore physical function, and its application has become widespread [1]. Because data in this field are being actively collected in Japan, nutritional knowledge is increasingly essential for rehabilitation professionals. In older adults, deficiencies have been reported not only in protein but also in micronutrients such as vitamin B1, niacin, iron, and sodium [2,3]. Because nutritional therapy alone is often insufficient to improve physical functions such as increasing muscle mass or enhancing gait speed in individuals with sarcopenia, combining it with exercise therapy is considered crucial. The International Conference on Frailty and Sarcopenia Research (ICFSR) reported that, although the level of evidence is low, combining nutritional and exercise therapies improved gait speed and knee extension strength more effectively than either intervention alone [4]. Sarcopenia is characterized by a loss of muscle mass secondary to a decline in muscle strength, which originates from nervous system dysfunction. In older adults, nutritional deficiencies, particularly in protein, can accelerate this loss of muscle strength and mass.

Recently, the concept of “respiratory sarcopenia” has been proposed and is gaining interest. Respiratory sarcopenia is diagnosed using indicators such as respiratory muscle strength (maximal expiratory pressure and maximal inspiratory pressure) and respiratory muscle mass measured using magnetic resonance imaging (MRI) or ultrasonography [5]. A lack of trunk muscle mass, which includes the respiratory muscles, is associated with poor spinal alignment [6]. Furthermore, maintaining the quality and quantity of trunk muscles contributes to a reduced risk of falls [7], suggesting a close relationship between trunk muscles and the physical function of older adults. We previously investigated the relationship between trunk muscle mass, measured using bioelectrical impedance analysis (BIA), and respiratory muscle strength [8]. Our results showed no correlation in the sarcopenic group, but a significant correlation was observed in the non-sarcopenic group. Based on this, we reported the importance of focusing on trunk muscles, including respiratory muscles, before the onset of sarcopenia.

Given the importance of rehabilitation nutrition for older adults, further verification of the effects of nutritional interventions is required, and the longitudinal effects of these interventions have not been sufficiently examined. Specifically, it remains unclear which body regions (upper limbs, lower limbs, or trunk) benefit in terms of muscle mass, which physical functions (such as gait speed or handgrip strength) improve, and at what point after the intervention these effects become apparent. The current study has the potential to provide insights into these questions.

Therefore, the primary purpose of this preliminary study was to investigate the longitudinal changes in trunk muscle mass following a combined nutritional and exercise intervention. The secondary purposes were to assess changes in upper and lower limb muscle mass, maximal gait speed, and handgrip strength at three and six months after the start of the intervention. By clarifying these longitudinal changes, we seek to obtain foundational data that can contribute to future large-scale studies.

## Materials and methods

Study design

This longitudinal study was conducted from January 2024 to June 2024, involving older adults attending a single daycare facility in a mid-sized city in Shiga, Japan. All participants were provided with a thorough explanation of the study and gave their written informed consent prior to participation. The study was conducted in compliance with the principles of the Declaration of Helsinki, and it was approved by the Kanazawa Orthopedic Surgery and Medical Clinic Ethics Committee (approval number: Kanazawa-OSMC-2023-004).

Participants

The study included 18 community-dwelling older adults (5 men, 13 women; height: 158.2 ± 9.3 cm; weight: 55.2 ± 15.0 kg; age: 80.4 ± 3.8 years; body mass index (BMI): 21.7 ± 4.0 kg/m^2^) who were users of the daycare service. All participants were certified as requiring support or long-term care under Japan’s long-term care insurance system. This national system classifies individuals into seven levels based on their physical and cognitive needs: two levels are designated as "requiring support," and five levels as "requiring long-term care." In this study, these were represented numerically as care levels 1-7, where a higher number indicates a greater need for assistance. All participants were able to walk independently or with a walking aid. Exclusion criteria were severe cognitive impairment and the presence of pain or significant postural abnormalities that affected weight-bearing or walking. All older adults at the daycare center who met the inclusion/exclusion criteria and provided their written informed consent during the study period were included in the study.

Intervention 

Participants attended the daycare facility once or twice a week (mean: 1.64 times/week). During each visit, they consumed a nutritional supplement drink (160 kcal energy, 11.0 g protein, 2.22 g fat, 24.0 g carbohydrates, 0.084-0.204 g salt equivalent, 200 mg calcium, 0.65 mg vitamin B1, 0.70 mg vitamin B2, 0.90 mg vitamin B6, 20.0 μg vitamin D), with 100% adherence to intake. Additionally, each participant received an individualized program that included resistance training for the lower limbs or trunk and exercise using a cycle ergometer. The frequency and intensity of the exercises were individually adjusted by a physical therapist who assessed the participant’s physical function and level of fatigue.

Measurements

All measurements were performed at three time points: baseline, three months, and six months.

Skeletal Muscle Mass

Skeletal muscle mass was measured using a BIA body composition analyzer (Inbody470; InBody Japan Inc., Tokyo, Japan). Prior to the analysis, all participants were asked to rest in a seated position for a sufficient period to ensure they were in a calm and stable state. During measurement, participants stood upright on the device with both feet, while a physical therapist provided support to ensure safety. The calculated upper limb, lower limb, and trunk muscle mass were used for the analysis.

Maximal Gait Speed

Maximal gait speed was measured on a 5m flat walkway within the facility. Participants started from a standing position and were instructed to walk as fast as possible. The time taken for the first foot to cross the start line until it passed the 5m line was measured with a stopwatch. To ensure safety, the measurement was taken under the supervision of the measurement staff after several practice walks. Gait speed (m/second) was calculated from the measured time and walking distance (5 m).

Handgrip Strength

Handgrip strength was measured in a standing position using a handgrip dynamometer (T.K.K.5401 GRIP-D; Takei Scientific Instruments Co., Ltd., Tokyo, Japan). Participants stood with their arms naturally at their sides. Under the supervision of the measurement staff, the formal measurement was conducted after several practice attempts, with care taken to avoid significant changes in posture. Measurements were taken for both the left and right hands, and the maximum value was used.

Statistical analysis

 All statistical analyses were performed using IBM SPSS Statistics for Windows, version 27 (IBM Corp., Armonk, New York, United States). The significance level was set at p < 0.05. A one-way repeated measures analysis of variance (ANOVA) was used to compare upper limb muscle mass, lower limb muscle mass, trunk muscle mass, gait speed, and handgrip strength across the three time points (baseline, three months, and six months). First, the normality of each variable was checked using the Shapiro-Wilk test. All variables except trunk muscle mass were found to be normally distributed (p ≥ 0.05). The assumption of sphericity was assessed using Mauchly's test. This assumption was violated for trunk muscle mass (p < 0.05), so the degrees of freedom were adjusted using the Greenhouse-Geisser correction. If the ANOVA results showed a significant difference, the Bonferroni method was used for post hoc multiple comparisons.

## Results

A total of 18 participants were included in the study. The baseline characteristics of the participants are summarized in Table 1. Descriptive statistics for each measurement item at each time point are shown in Table 2.

**Table 1:** Baseline characteristics of the participants (N=18) *Care level is based on Japan's Long-Term Care Insurance system, ranging from 1 (lowest support need) to 7 (highest care need).

Variables	Values
Age (years), mean±SD	80.4±3.8
BMI (kg/m²), mean±SD	21.7±4.0
Weight (kg), mean±SD	55.2±15.0
Height (cm), mean±SD	158.2±9.3
Care level*, n	
Level 1	5
Level 2	3
Level 3	6
Level 4	4
Level 5～7	0

**Table 2:** Descriptive values for outcome measures at each time point

Variables	Baseline, mean±SD	3 months, mean±SD	6 months, mean±SD
Upper limb muscle mass (kg)	3.8±1.3	3.9±1.2	4.0±1.2
Lower limb muscle mass (kg)	11.5±2.7	11.6±2.8	11.7±2.7
Trunk muscle mass (kg)	17.1±4.3	17.5±3.8	17.7±3.8
Gait speed (m/s)	0.9±0.3	0.9±0.3	1.0±0.3
Handgrip strength (kg)	21.1±8.0	21.2±7.5	21.5±8.1

The results of the repeated measures one-way ANOVA are summarized in Table 3. According to the Shapiro-Wilk test, all variables were normally distributed except for trunk muscle mass. For trunk muscle mass, the assumption of sphericity was violated, and the Greenhouse-Geisser correction was therefore applied. For all other variables, the assumption of sphericity was met.

**Table 3:** Results of repeated measures ANOVA for the main effect of time *For trunk muscle mass, values are based on the Greenhouse-Geisser correction as the assumption of sphericity was violated.

Variables	F	df	ｐ	η_p_^2^
Upper limb muscle mass	3.859	2, 34	0.031	0.185
Lower limb muscle mass	0.619	2, 34	0.545	0.035
Trunk muscle mass*	5.896	1.5, 25.5	0.013	0.258
Gait speed	0.377	2, 34	0.689	0.022
Handgrip strength	0.301	2, 34	0.742	0.017

As shown in Table 2, gradual increases were observed over the six-month period for trunk muscle mass (from 17.1±4.3 kg to 17.7±3.8 kg) and upper limb muscle mass (from 3.8±1.3 kg to 4.0±1.2 kg). The analysis of these changes, summarized in Table 3, revealed a significant change over time for trunk muscle mass (F (1.5, 25.5) = 5.896, p = 0.013, ηp2 =0.258) and upper limb muscle mass (F (2, 34) = 3.859, p = 0.031, ηp2 =0.185). In contrast, no significant change over time was found for lower limb muscle mass, gait speed, or handgrip strength.

Post hoc tests with Bonferroni correction were performed for the variables that showed a significant main effect. The results are shown in Table 4. For trunk muscle mass, a significant increase was observed at six months after the start of intervention compared to baseline (mean difference = 0.778 kg, p = 0.043). No significant differences were found between baseline and three months or between three months and six months (p > 0.05).

**Table 4:** Pairwise comparisons for trunk and upper limb muscle mass using Bonferroni post hoc tests. *p<0.05

Point of time	Baseline, mean difference (p-value)	3 months, mean difference (p-value)	6 months, mean difference (p-value)
Trunk muscle mass			
Baseline	-	-0.472 (0.114)	-0.778 (0.043)^＊^
3 months	0.472 (0.114)	-	-0.306 (0.302)
6 months	0.778 (0.043)^＊^	0.306 (0.302)	-
Upper limb muscle mass			
Baseline	-	-0.139(0.298)	-0.206(0.087)
3 months	0.139(0.298)	-	-0.067(0.793)
6 months	0.206(0.087)	0.067(0.793)	-

For upper limb muscle mass, the Bonferroni-corrected post hoc tests revealed no significant pairwise differences between any of the time points (all p > .05). The comparison between baseline and six months post the start of intervention showed the largest difference, although it did not reach statistical significance (mean difference = 0.206 kg, p =0.087).

## Discussion

The primary result of this preliminary study is that a six-month intervention combining a nutritional supplement drink with an individualized exercise program led to a significant increase in trunk muscle mass among older adults attending daycare. Upper limb muscle mass also showed a significant overall change over the intervention period; however, unlike trunk muscle mass, post-hoc analysis with Bonferroni correction did not reveal a significant difference between any two time points. In contrast, changes in lower limb muscle mass did not reach statistical significance. The finding that only trunk muscle mass changed significantly is a noteworthy insight for rehabilitation nutrition.

Trunk muscles, such as the abdominal and back muscle groups, have been reported to contribute to postural stability and balance and are considered clinically important [9-11]. For instance, previous studies have reported that trunk muscle strengthening improves balance in older adults [9,10] and that the muscle thickness of specific trunk muscles (e.g., rectus abdominis, internal oblique) and the quadriceps are determinants of balance [11]. In light of this background, clarifying how trunk muscle mass changes in response to an intervention is clinically important.

In the diagnosis of sarcopenia in older adults, muscle mass assessment has primarily relied on appendicular skeletal muscle mass measured by BIA or dual-energy X-ray absorptiometry (DXA) [12-14]. While trunk muscle mass is gaining attention for its connection to respiratory function [5,15], it has not been emphasized as an outcome measure for nutritional and exercise interventions. The novelty of this study lies in demonstrating that a combined nutrition and exercise intervention is effective for increasing trunk muscle mass. The responsiveness of trunk muscles to this intervention may be attributable to their histological characteristics and activity patterns.

The abdominal muscles are composed of a mixture of diverse muscle fiber types [16], while the erector spinae group is particularly rich in type I (slow-twitch) fibers, which are involved in sustained posture maintenance [17,18]. These muscles are continuously or frequently active to maintain posture and stabilize the trunk in daily life. Furthermore, the main respiratory muscle groups involved in breathing movements are also located in the trunk. Therefore, it is possible that these muscles, which have a higher frequency of activity compared with limb muscles, are more responsive to combined interventions like the one in the current study. The results of this study suggest the importance of holistic intervention strategies focusing on trunk function for sarcopenia and frailty prevention in older adults, in addition to the conventional limb-centric approach. From the perspective of the recently proposed condition of respiratory sarcopenia, improving trunk muscle mass may contribute to better respiratory function and quality of life, warranting further research.

The nutritional supplement drink used in this study provided 160 kcal of energy and 11.0 g of protein per serving, along with other vitamins and minerals. Considering the recommended protein intake for older adults, especially those with chronic diseases or high activity levels (≥ 1.2 g/kg/day) [19], this supplement was likely an effective means of providing additional protein to the participants, who were certified for long-term care. Consuming an amount exceeding the general recommendation of 0.8 g/kg/day [20], in conjunction with exercise, is thought to have contributed to the results. While combining nutrition and exercise is effective for maintaining and increasing muscle mass, practical challenges exist. Older adults tend to have lower energy intake compared to younger individuals [21], and securing adequate nutrition from meals alone can increase the volume of food and the financial burden [22,23]. The supplement used in this study is highly practical, requiring no preparation, being easy to consume, and containing protein and other micronutrients, thereby likely enhancing compliance and promoting continued use. Dietary diversity tends to decrease in old age, a factor that has been linked to the progression of frailty [24,25]. The multi-nutrient composition of this drink may have also been beneficial in this regard.

Limitations

This study has several limitations. First, the sample size was small, consisting of 18 participants from a single daycare facility, and their backgrounds were relatively homogeneous. Therefore, the generalizability of these findings is limited. Furthermore, a small sample size can lead to insufficient statistical power, particularly after applying conservative corrections for multiple comparisons. For upper limb muscle mass, although the post-hoc tests did not reveal a significant pairwise difference, the overall analysis showed a large effect size (ηp2 = 0.185). This suggests that a clinically relevant increase likely occurred and that the lack of statistical significance in the post-hoc analysis is probably due to the study's limited statistical power. This finding highlights a promising area for future research with larger sample sizes. In contrast to the strong effects observed for the trunk and upper limbs, the intervention's effects on lower limb muscle mass, gait speed, and handgrip strength were not statistically significant. The small effect sizes associated with these outcomes (all ηp2 < 0.04) suggest that this was not merely due to a lack of statistical power, but rather that the intervention, as designed, had a genuinely limited impact on these specific measures. This finding provides clarity on the specificity of the program's benefits, primarily targeting the trunk and upper body musculature. Second, this study did not include a control group. Thus, it is difficult to clearly determine whether the observed significant increase in trunk muscle mass was a direct effect of the combined nutritional and exercise intervention or attributable to the passage of time or other factors. Third, the intensity and frequency of the exercise program were individually adjusted by a physical therapist on the basis of each participant's physical function and condition on a given day. While this individualized approach is clinically appropriate, the lack of a standardized program presents challenges for the reproducibility of the intervention and its comparability with other studies.

In light of these limitations, the current study should be considered as a preliminary investigation into the longitudinal effects of rehabilitation nutrition on trunk muscle mass. Future research should aim to conduct randomized controlled trials with larger, more diverse populations and a control group. Furthermore, it is necessary to more rigorously validate the effectiveness of rehabilitation nutrition by controlling for potential confounding factors, such as daily dietary content and physical activity levels, and by using a standardized intervention protocol.

## Conclusions

This preliminary study indicated that a six-month combined nutritional and exercise intervention can lead to a significant increase in trunk muscle mass in older adults requiring daycare. Given that trunk muscles are clinically vital for postural stability, balance, and respiratory function, and that their responsiveness to such interventions has been under-investigated, our findings are noteworthy. They highlight the potential importance of including the trunk as a key target in rehabilitation nutrition strategies. We therefore conclude that focusing on trunk muscles, in addition to limb muscles, is a valuable approach that warrants further investigation in larger, controlled studies for the management of sarcopenia and frailty.
